# Sohlh1 Modulates the Stemness and Differentiation of Glioma Stem‐Like Cells by Inactivation of Wnt/β‐Catenin Signalling Pathway via SFRP1

**DOI:** 10.1111/jcmm.70599

**Published:** 2025-05-26

**Authors:** Sujuan Zhi, YanRu Chen, Yunling Xiao, Lanlan Liu, Xiaoning Feng, Xuyue Liu, Ying Shen, RuiHong Zhang, Jing Hao

**Affiliations:** ^1^ Key Laboratory of the Ministry of Education for Experimental Teratology, Department of Histology and Embryology, School of Basic Medical Sciences, Cheeloo College of Medicine Shandong University Jinan Shandong People's Republic of China; ^2^ Liver Transplantation Center, National Clinical Research Center for Digestive Diseases, Beijing Friendship Hospital Capital Medical University Beijing China; ^3^ Department of Geriatric Medicine, Qilu Hospital, Cheeloo College of Medicine Shandong University Jinan Shandong People's Republic of China

**Keywords:** differentiation, glioma stem‐like cells, SFRP1, Sohlh1, stemness

## Abstract

Glioma stem‐like cells (GSLCs) are tumour initiating cells that drive tumorigenesis and progression through self‐renewal and various differentiation potencies. Therefore, the identification of genes required for GSLC stemness and differentiation is vital for the development of novel targeted therapies. Sohlh1 belongs to the superfamily of bhlh transcription factors and serves as a tumour suppressor in glioma. The role of Sohlh1 in GSLCs remains unknown. Here, we demonstrated that Sohlh1 functioned through the SFRP1/Wnt/β‐catenin signalling pathway and led to the consequent suppression of GSLC stemness and the promotion of GSLC differentiation in vitro and in vivo. Moreover, Sohlh1 could directly bind to the promoter of SFRP1 and promote its transcriptional activity. SFRP1 mediated the effects of Sohlh1 on GSLC stemness and differentiation. Clinically, we observed a significant inverse correlation between Sohlh1 and Nestin expression levels, and a positive correlation between Sohlh1 and SFRP1 expression in glioma tissues. Collectively, our findings suggest an important role of the Sohlh1/SFRP1/Wnt/β‐catenin pathway in regulating GSLC stemness and differentiation, suggesting potential therapeutic targets for glioma.

## 1 Introduction

Glioma is the most common intracranial malignant brain tumour [1], and one of the major causes of human mortality [2, 3]. Combining histological and molecular characteristics, in 2021 the World Health Organisation (WHO) classified glioma into four grades: 1, 2, 3, and 4 [4]. Among them, grades 1, 2, and 3 are called low‐grade glioma (LGG), whereas grade 4 is called high‐grade glioma (HGG) [5, 6]. Glioblastoma, IDH‐wildtype is the most malignant of all gliomas and belongs to grade 4 [7]. It has a postdiagnosis lifespan of 14.6 months and a 1‐year survival rate of only 10% [8]. Currently, although the treatment of glioma has made great progress in surgery, radiotherapy, chemotherapy and targeted therapy, the clinical outcome is still not satisfactory [9]. Many glioma patients suffer from recurrence even after standard combined treatment. Therefore, elucidating the molecular mechanism of glioma recurrence remains imperative to improve the prognosis of glioma patients.

Cancer stem‐like cells (CSLCs) are a small subset of cells in tumour tissues and are characterised by the self‐renewal capacity, unlimited division and multi‐directional differentiation [10, 11]. CSLCs are considered to be the major cause of treatment failure [12]. The existence of CSLCs has been demonstrated in various types of tumours, including glioma [13, 14]. Glioma stem‐like cells (GSLCs) can be labelled by stem‐like cell markers, such as Nestin, CD133, CD44, SOX2, OCT4, CD15, A2B5, CD90, ITGA6, CD171, S100A4 [15]. Several major signalling pathways are involved in the regulation of stemness and differentiation of GSLCs, including Wnt, Notch and Hedgehog signalling [16, 17, 18, 19].

Wnt/β‐catenin signalling pathway is an evolutionarily highly conserved pathway that regulates cell proliferation, migration and apoptosis, and is involved in the regulation of embryonic development, tissue homeostasis, neurogenesis and the development of many human diseases [20, 21]. Wnt signalling is triggered by active Wnt ligands binding to their membrane receptors frizzled (FZD) and LDL receptor‐related protein 5 or 6 (LRP5/6), which leads to dissociation of β‐catenin from the ‘destruction complex’ containing of Axin, APC, CK1α and GSK‐3β [16, 21]. Consequently, cytosolic β‐catenin translocates to the nucleus to bind to transcription factors, LEF/TCF, and thus induces the transcription of various downstream genes, such as C‐myc and Cyclin D1 [22, 23]. Numerous studies have shown that the Wnt/β‐catenin signalling pathway is hyperactivated in GSLCs and promotes tumour growth, invasion, radiotherapy resistance and recurrence by maintaining the stemness of GSLCs in glioma [24, 25]. The initiation process of Wnt ligand binding to receptors can be directly blocked by extracellular antagonistic proteins, including SFRP1, DKK1, DKK3, etc.

Secreted Frizzled‐related protein 1 (SFRP1), a member of the SFRP family, represses Wnt/β‐catenin signalling by directly binding Wnt ligands and sequestering them away from FZD receptors [26, 27]. SFRP1 has been identified as a tumour suppressor gene in glioma, silenced by aberrant promoter methylation and loss of function mutations [28]. In addition to epigenetic regulation, the modulation mechanism of SFRP1 expression in GSLCs remains unclear.

Sohlh1 belongs to the superfamily of basic helix–loop–helix (bhlh) transcription factors. Our previous results have confirmed that Sohlh1 is a novel tumour suppressor in glioma. Forced Sohlh1 expression suppresses cancer cell proliferation via inhibition of the Wnt/β‐catenin signalling pathway [29]. Preliminary results showed that Sohlh1 expression in GSLCs was significantly down‐regulated, suggesting that Sohlh1 might play an important role in the regulation of GSLC stemness and differentiation. Therefore, we hypothesised that Sohlh1 may be involved in the regulation of stemness and differentiation of GSLCs by inhibiting the Wnt/β‐catenin signalling pathway.

In this study, Sohlh1 was functionally analysed in GSLCs. Sohlh1 suppressed GSLC stemness, whilst promoting GSLC differentiation. We demonstrated that Sohlh1 was a key regulator of SFRP1/Wnt/β‐catenin signalling in GSLCs. In addition, Sohlh1 was positively correlated with the expression of SFRP1, whereas negatively associated with the expression of Nestin in glioma tissues, suggesting a tumour suppressive role in the stemness of glioma.

## 2 Results

### 2.1 Sohlh1 Is Downregulated in Glioma and Predicts Good Prognosis

**FIGURE 1:**
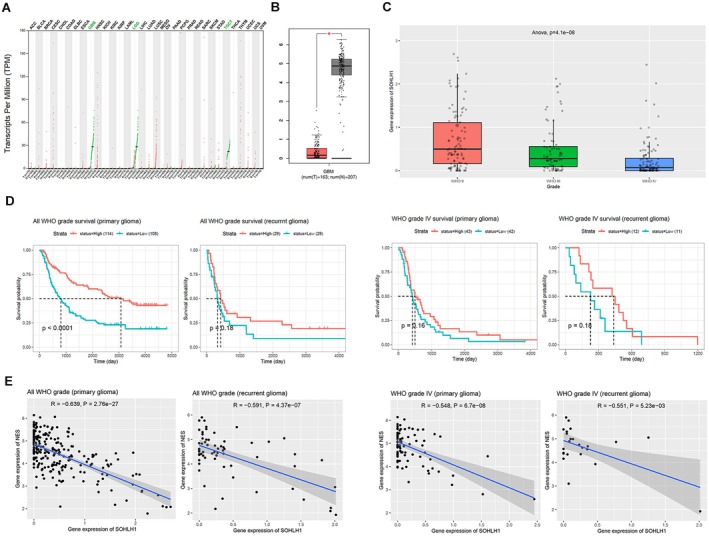
Sohlh1 is downregulated in glioma and predicts good prognosis. (A) Expression of Sohlh1 in different cancers based on the GEPIA database, red for tumour tissues and green for normal tissues. (B) Relative expression levels of Sohlh1 in glioma (left) and normal tissues (right) from CGGA database (in mRNASeq_−_325). (C) The expression of Sohlh1 in glioma tissues based on WHO classification from CGGA database. (D) Relevance of Sohlh1 expression levels to overall patient survival in all gliomas versus WHO grade IV gliomas according to CGGA database. (E) Correlation between Sohlh1 and NES (Nestin) expression levels in all gliomas and WHO grade IV gliomas according to CGGA database (in mRNASeq_−_325).

To elucidate the expression of Sohlh1 in glioma, we examined the transcriptional and survival data of Sohlh1 in the patients with glioma from the GEPIA (http://gepia.cancer‐pku.cn/) and CGGA databases (http://www.cgga.org.cn/) (in mRNASeq_−_325). The results showed that the expression of Sohlh1 was lower in glioma compared with the normal brain tissues (Figure 1A,B), and the expression of Sohlh1 decreased with increasing tumour malignancy (Figure 1C). Consistently, the overall survival of the glioma patients with high levels of Sohlh1 was higher than that with low expression of Sohlh1 (Figure 1D). These results suggested that Sohlh1 was lowly expressed in glioma and associated with a good prognosis. In addition, the analytic data from the CGGA database (in mRNASeq‐325) also showed that Sohlh1 expression in glioma tissues was negatively correlated with the expression of Nestin, a stemness marker (Figure 1E).

### 2.2 Sohlh1 Represses the Stemness of GSLCs

**FIGURE 2:**
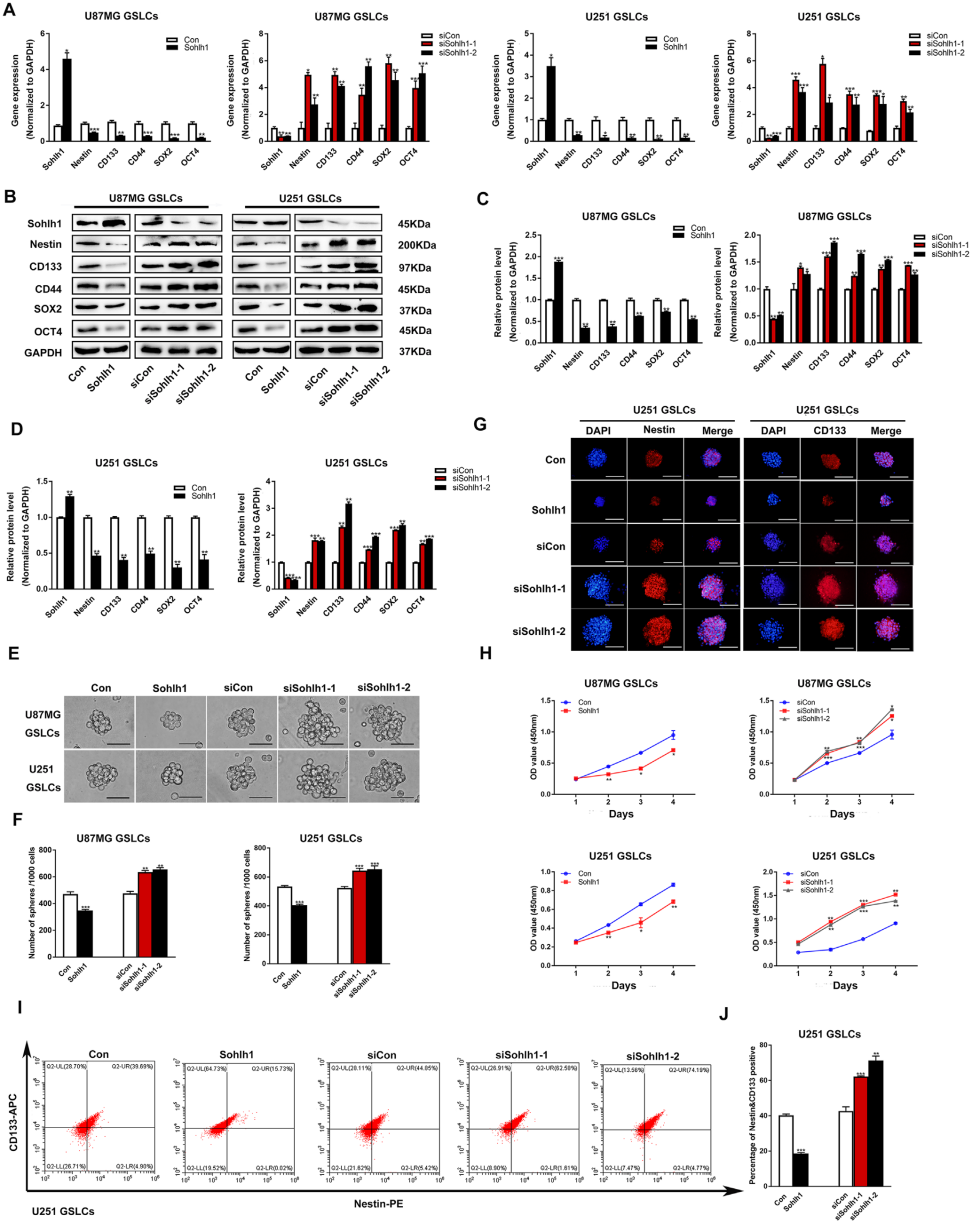
Sohlh1 represses the stemness of GSLCs. q‐PCR (A) and Western‐blot (B, C, D) were performed to detect the expression of stemness‐related genes, including Nestin, CD133, CD44, SOX2 and OCT4 in Sohlh1 overexpressing/knockdown GSLCs. (E, F) Neurosphere assay was performed to evaluate the stemness of GSLCs. Representative images and numbers of spheroids formed by Sohlh1‐overexpressing and Sohlh1‐knockdown U87MG and U251 GSLCs. Scale bars indicate 50 μm. (G) Representative IF images of Nestin and CD133 in U251 GSLC spheroids. Scale bars indicate 100 μm. (H) CCK‐8 assay was performed to examine cell viability in Sohlh1‐overexpressing and Sohlh1‐knockdown GSLCs. (I, J) Flow cytometry was performed to detect the effect of Sohlh1 on the proportion of Nestin and CD133 double positive GSLCs. Statistical results were calculated using a two‐tailed Student's *t* test. *p < 0.05, **p < 0.01, ***p < 0.001.

We cultured glioma cells U87MG and U251 in vitro and constructed Sohlh1 overexpression and Sohlh1 knockdown stable cell lines by lentivirus transfection. GSLCs were successfully enriched in low adhesion dishes with serum‐free medium. q‐PCR, Western‐blot, neurosphere formation, CCK‐8, immunofluorescence staining and FACS were performed to analyse the effects of Sohlh1 on the stemness of GSLCs.

q‐PCR and Western‐blot analysis showed that the mRNA and protein expression levels of stemness‐related genes, including Nestin, CD133, CD44, SOX2 and OCT4, were remarkably downregulated in Sohlh1 overexpression GSLCs, whereas the expression levels of the above‐mentioned genes were remarkably upregulated in the Sohlh1 knockdown groups, compared with the si‐control group (Figure 2A–D).

Moreover, the results of the neurosphere formation assay showed that the size and the number of GSLC neurospheres in the Sohlh1 group were smaller and less than those of the control group, whereas the results of the knockdown Sohlh1 were opposite (Figure 2E,F). The results of spheroid immunofluorescence staining showed relatively weak expression of stemness markers, such as Nestin and CD133 in the Sohlh1 groups (Figure 2G). The CCK8 assay showed that Sohlh1 markedly inhibited the viability of GSLCs, whereas the results were opposite in the Sohlh1 RNAi groups (Figure 2H). Consistently, the FACS results showed that the number of Nestin and CD133 double‐positive GSLCs was decreased in Sohlh1 overexpression GSLCs (Figure 2I,J). Taken together, these data suggest that Sohlh1 suppresses the stemness of GSLCs.

### 2.3 Sohlh1 Promotes the Differentiation of GSLCs

**FIGURE 3:**
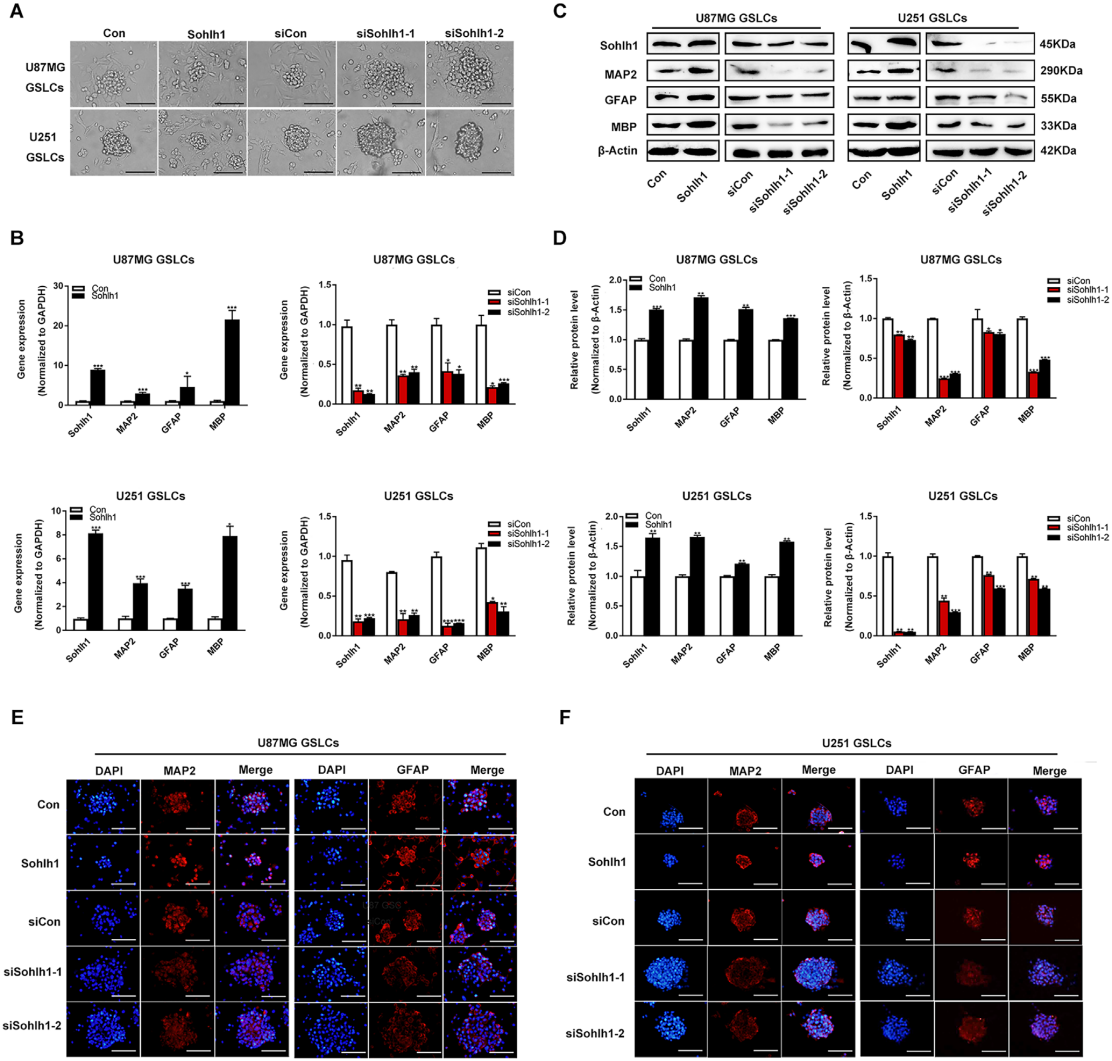
Sohlh1 promotes the differentiation of GSLCs. (A) U87MG and U251 GSLCs were cultured in medium containing 1% serum for 8 h. Representative images of induced differentiation of Sohlh1 overexpression and Sohlh1 knockdown in U87MG and U251 GSLCs. Scale bars indicate 100 μm. q‐PCR (B) and Western‐blot (C, D) were performed to detect the expression of GSLC differentiation‐related genes, including MAP2, GFAP and MBP. (E, F) Representative IF images of MAP2 and GFAP in differentiated spheroids of Sohlh1 overexpression and Sohlh1 knockdown in U87MG and U251 GSLCs. Scale bars indicate 100 μm. Statistical results were calculated using a two‐tailed Student's *t* test. *p < 0.05, **p < 0.01, ***p < 0.001.

To investigate whether Sohlh1 affects the differentiation of GSLCs, we performed an induction of differentiation assay with 1% serum and examined the expression levels of differentiation‐related markers of GSLCs, including microtubule‐associated protein 2 (MAP2), glial fibrillary acidic protein (GFAP), and myelin basic protein (MBP). Sohlh1 overexpression significantly promoted the differentiation of GSLC neurospheres and the expression of these differentiation‐related genes at mRNA and protein levels, whereas Sohlh1 knockdown repressed the expression of these differentiation‐related genes in U87MG and U251 GSLCs (Figure 3). Overall, these data demonstrate that Sohlh1 plays a key role in the differentiation of GSLCs.

### 2.4 Sohlh1 Suppresses the Growth of Intracranial Xenografts From GSLCs In Vivo

**FIGURE 4:**
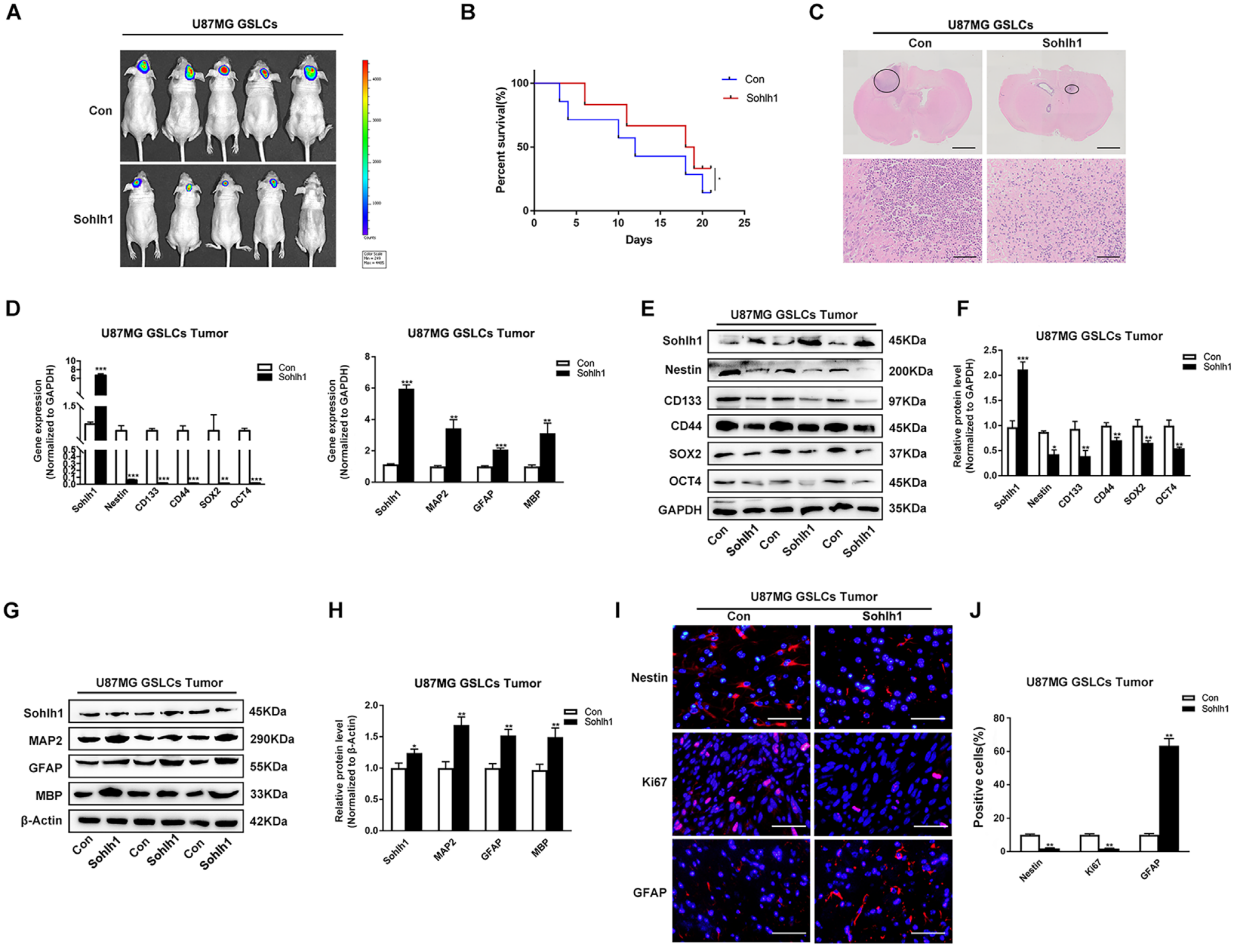
Sohlh1 suppresses the growth of intracranial xenografts from GSLCs in vivo. (A) 1 × 10^6^ Sohlh1‐overexpressing U87MG GSLCs were injected intracranially in nude mice. Representative bioluminescence images on day 21 after injection. (B) Kaplan–Meier survival curves in nude mice of intracranial xenograft model. (C) Representative images of the coronal sections of mouse brain harvested on day 21 after injection are shown. q‐PCR (D) and Western‐blot (E, F) analysis of the expression of GSLC stemness markers, including Nestin, CD133, CD44, SOX2 and OCT4 in xenografts tissues. q‐PCR (D) and Western‐blot (G, H) analysis of the expression levels of GSLC differentiation markers, including MAP2, GFAP and MBP in xenografts tissues. (I, J) Representative IF images of Nestin, Ki67, GFAP in xenografts tissues. Bar graphs show the statistical data for the positive cells. Scale bars indicate 50 μm. Statistical results were calculated using a two‐tailed Student's *t* test. *p < 0.05, **p < 0.01, ***p < 0.001.

To examine the effects of Sohlh1 on the stemness and differentiation of GSLCs in vivo, the Con and Sohlh1 overexpressing U87MG‐Luc GSLCs were injected into the frontal cortex of nude mice brain to construct an intracranial xenograft model. Bioluminescence imaging results showed that overexpression of Sohlh1 inhibited intracranial tumour growth and improved the survival of nude mice compared to the control group (Figure 4A–C). Besides, the expression of Nestin, CD133, CD44, SOX2 and OCT4 was significantly downregulated at mRNA and protein levels, whereas the expression levels of MAP2, GFAP and MBP were upregulated in Sohlh1‐overexpressing GSLC‐derived xenografts (Figure 4D–H).

The results of immunofluorescence staining in brain tissue sections indicated a decrease in the expression of the GSLC marker Nestin and the proliferation marker Ki67, and an increase in the differentiation marker GFAP in the Sohlh1 group (Figure 4I,J). Taken together, these results suggest that Sohlh1 markedly restrains the growth of intracranial tumours by controlling the stemness and differentiation of GSLCs in vivo.

### 2.5 Sohlh1 Inhibits the Activation of Wnt/β‐Catenin Signalling Pathway

**FIGURE 5:**
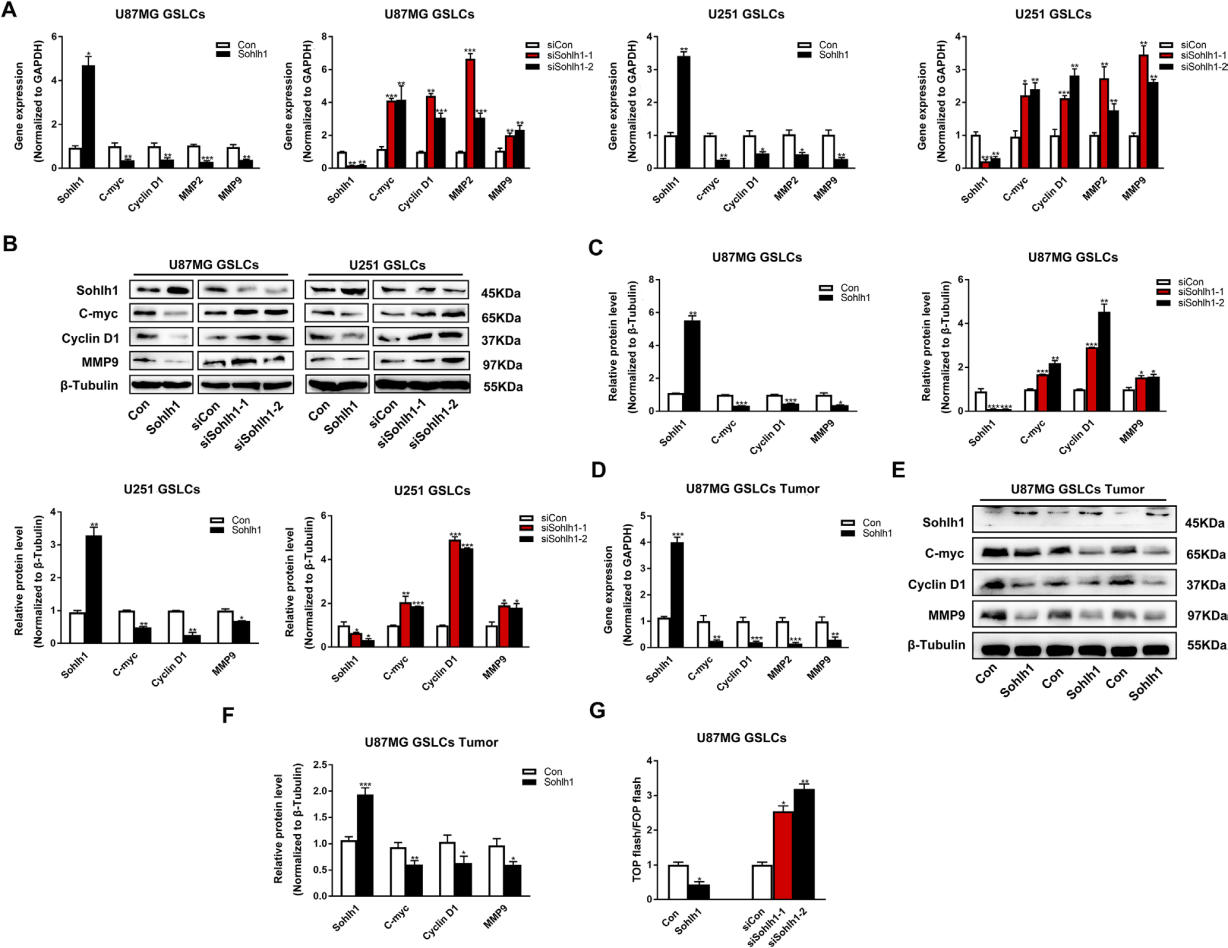
Sohlh1 inhibits Wnt/β‐catenin signalling pathway activation. (A) q‐PCR analysis for mRNA levels of C‐myc, Cyclin D1, MMP2, MMP9 in Sohlh1 overexpressing and Sohlh1 knockdown U87MG/U251 GSLCs. (B, C) Western‐blot analysis for protein levels of C‐myc, Cyclin D1, MMP9 in Sohlh1 overexpressing and Sohlh1 knockdown U87MG/U251 GSLCs. (D) q‐PCR analysis for mRNA levels of C‐myc, Cyclin D1, MMP2, MMP9 in tumour tissues. (E, F) Western‐blot analysis for protein levels of C‐myc, Cyclin D1, MMP9 in tumour tissues. (G) TOP/FOP luciferase activity to assess the activation of Wnt/β‐catenin signalling in Sohlh1 overexpressing and Sohlh1 knockdown U87MG GSLCs. Statistical results were calculated using a two‐tailed Student's *t* test. *p < 0.05, **p < 0.01, ***p < 0.001.

Wnt/β‐catenin signalling pathway plays a major role in stemness maintenance and differentiation of tumour stem‐like cells [17, 20]. To investigate the mechanism of Sohlh1 in stemness and differentiation of GSLCs, q‐PCR and Western‐blot assays were performed to detect the expression of downstream target genes of the Wnt/β‐catenin signalling pathway in U87MG and U251 GSLCs. As shown in Figure 5A–C, Sohlh1 significantly suppressed the expression of C‐myc, Cyclin D1, MMP2 and MMP9 at mRNA and protein levels, whereas knockdown of Sohlh1 promoted the expression of these genes. Consistently, similar results were obtained from intracranial xenografts (Figure 5D–F). In addition, we transfected TOP flash and FOP flash plasmids into U87MG GSLCs; luciferase report assay showed that overexpression of Sohlh1 decreased the ratio of TOP flash/FOP flash luciferase activity, whereas knockdown of Sohlh1 increased the ratio of TOP flash/FOP flash luciferase activity (Figure 5G). To sum up, these results demonstrate that Sohlh1 inhibits Wnt/β‐catenin signalling pathway activation in GSLCs.

### 2.6 Sohlh1 Up‐Regulates the Expression of SFRP1 in GSLCs

**FIGURE 6:**
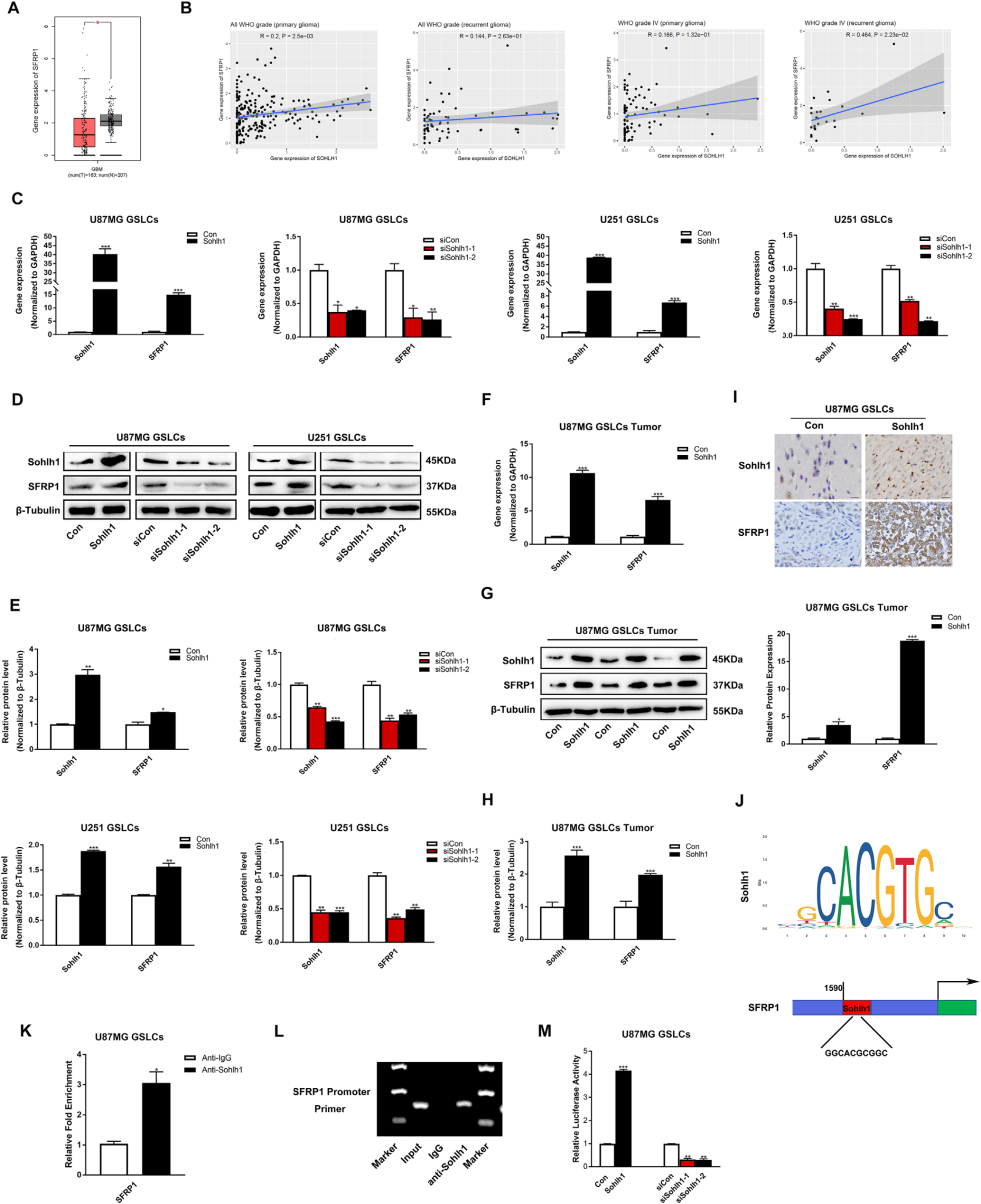
Sohlh1 up‐regulates the expression of SFRP1 in GSLCs. (A) Expression of SFRP1 in normal tissues (left) and glioma tissues (right) from TCGA database. (B) Correlation between Sohlh1 and SFRP1 expression levels in gliomas based on CGGA database (http://www.cgga.org.cn/) (in mRNASeq_−_325). q‐PCR analysis for mRNA levels of SFRP1 in GSLCs (C) and tumour tissues (F). Western‐blot analysis for protein levels of SFRP1 in GSLCs (D, E) and tumour tissues (G, H). (I) Representative IHC images of Sohlh1 and SFRP1 in tumour tissues. Scale bars indicate 20 μm. (J) Potential binding sites of Sohlh1 in SFRP1 promotor were predicted by JASPAR. (K, L) ChIP analysis of forced Sohlh1 expression U87MG GSLCs using anti‐Sohlh1 antibody for SFRP1 promoter. (M) Luciferase report assay was performed to detect the effecf of Sohlh1 on transcriptional activity of SFRP1 in U87MG GSLCs. Statistical results were calculated using a two‐tailed Student's *t* test. *p < 0.05, **p < 0.01, ***p < 0.001.

Recent studies have demonstrated that SFRP1, an antagonist of Wnt/β‐catenin signalling, represses the maintenance of GSLC stemness [27]. To investigate whether Sohlh1 inhibits Wnt/β‐catenin signalling pathway activation via regulation of SFRP1 expression, The TCGA database analysis showed that SFRP1 expression was significantly lower in glioma than in normal tissues (Figure 6A), and the CGGA database analysis showed a positive correlation between Sohlh1 and SFRP1 expression in glioma (Figure 6B). Next, we examined the effects of Sohlh1 on SFRP1 expression in GSLCs and GSLC‐derived tumour tissues. The q‐PCR, Western‐blot and IHC results showed that overexpression of Sohlh1 elevated SFRP1 expression at mRNA and protein levels, whereas knockdown of Sohlh1 obtained the opposite effects (Figure 6C–I). To further elucidate the molecular mechanism of Sohlh1 regulation of SFRP1 expression, we predicted the binding sites of Sohlh1 to the SFRP1 promoter using the JASPAR database; the results of the ChIP assay revealed that Sohlh1 could directly bind to the promoter region of SFRP1 (Figure 6J–L). In the luciferase report assay, our data indicated that Sohlh1 overexpression elevated the luciferase activity driven by the Luc‐SFRP1 promoter, whereas Sohlh1 knockdown suppressed the luciferase activity (Figure 6M). Collectively, our results suggest that SFRP1 is a direct target of Sohlh1.

### 2.7 SFRP1 Mediates the Effects of Sohlh1 in GSLCs

**FIGURE 7:**
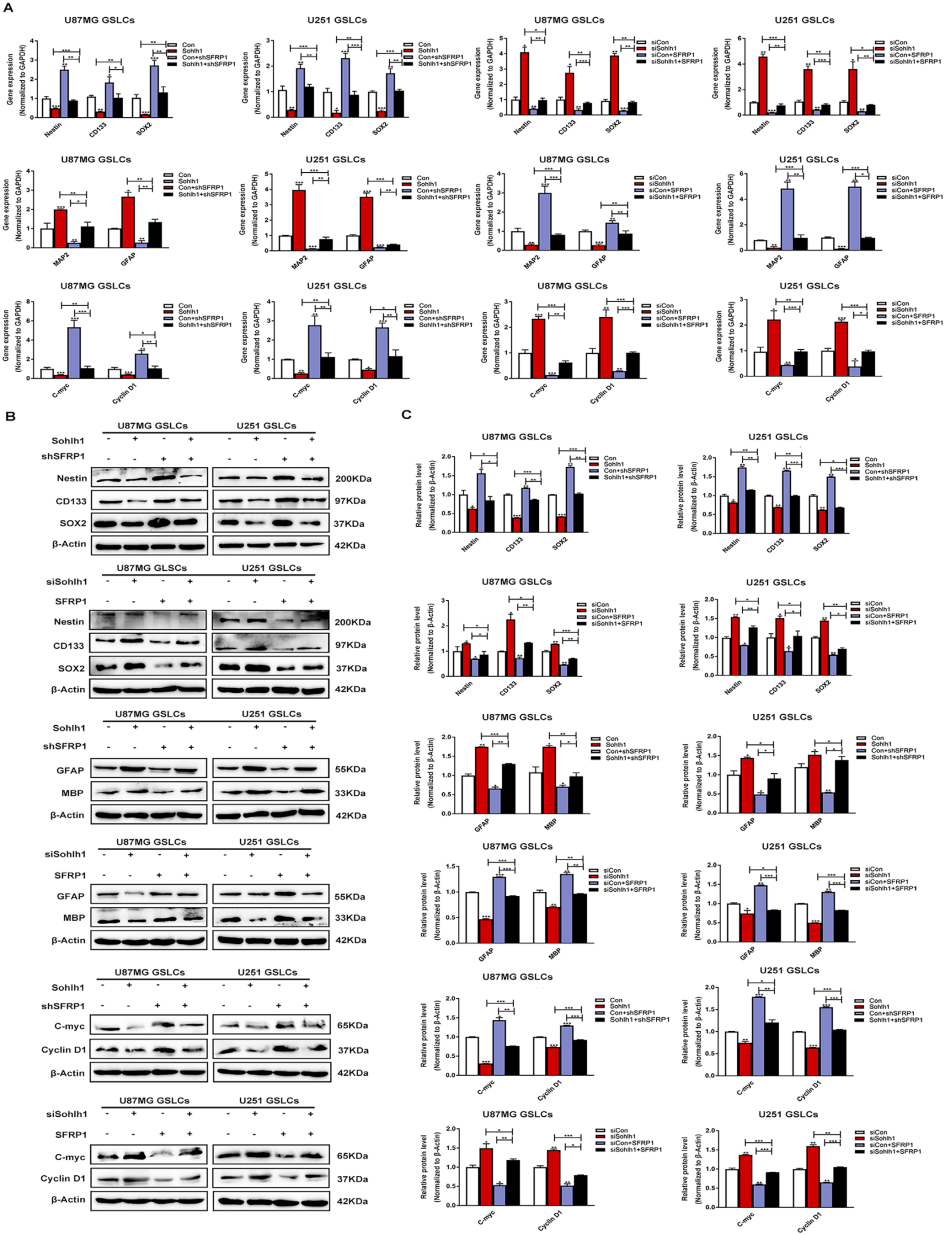
SFRP1 mediates the effects of Sohlh1 in GSLCs. SFRP1 knockdown/overexpression plasmids were transfected into Sohlh1 overexpression/knockdown GSLCs. q‐PCR (A) and Western‐blot (B, C) were used to detect the expression levels of the stemness markers Nestin, CD133, SOX2, the differentiation markers MAP2, GFAP and the downstream target genes C‐myc and Cyclin D1 of the Wnt/β‐catenin signalling pathway. Statistical results were calculated using a two‐tailed Student's *t* test. *p < 0.05, **p < 0.01, ***p < 0.001.

To further investigate whether SFRP1 mediates the impacts of Sohlh1 in GSLCs, we transfected SFRP1 knockdown/overexpression plasmids in Sohlh1 overexpressing/knockdown GSLCs. The results of q‐PCR and Western‐blot analysis showed that SFRP1 could partially attenuate the effects of Sohlh1 on the expression of stemness‐related markers, differentiation‐related genes and the downstream target genes of the Wnt/β‐catenin signalling pathway in GSLCs (Figure 7). In conclusion, these data suggest that Sohlh1 regulates the stemness and differentiation of GSLCs via the upregulation of SFRP1 expression.

### 2.8 Sohlh1 Is Negatively Correlated With the Expression of Nestin and Positively Correlated With the Expression of SFRP1 in Glioma Tissues

**FIGURE 8:**
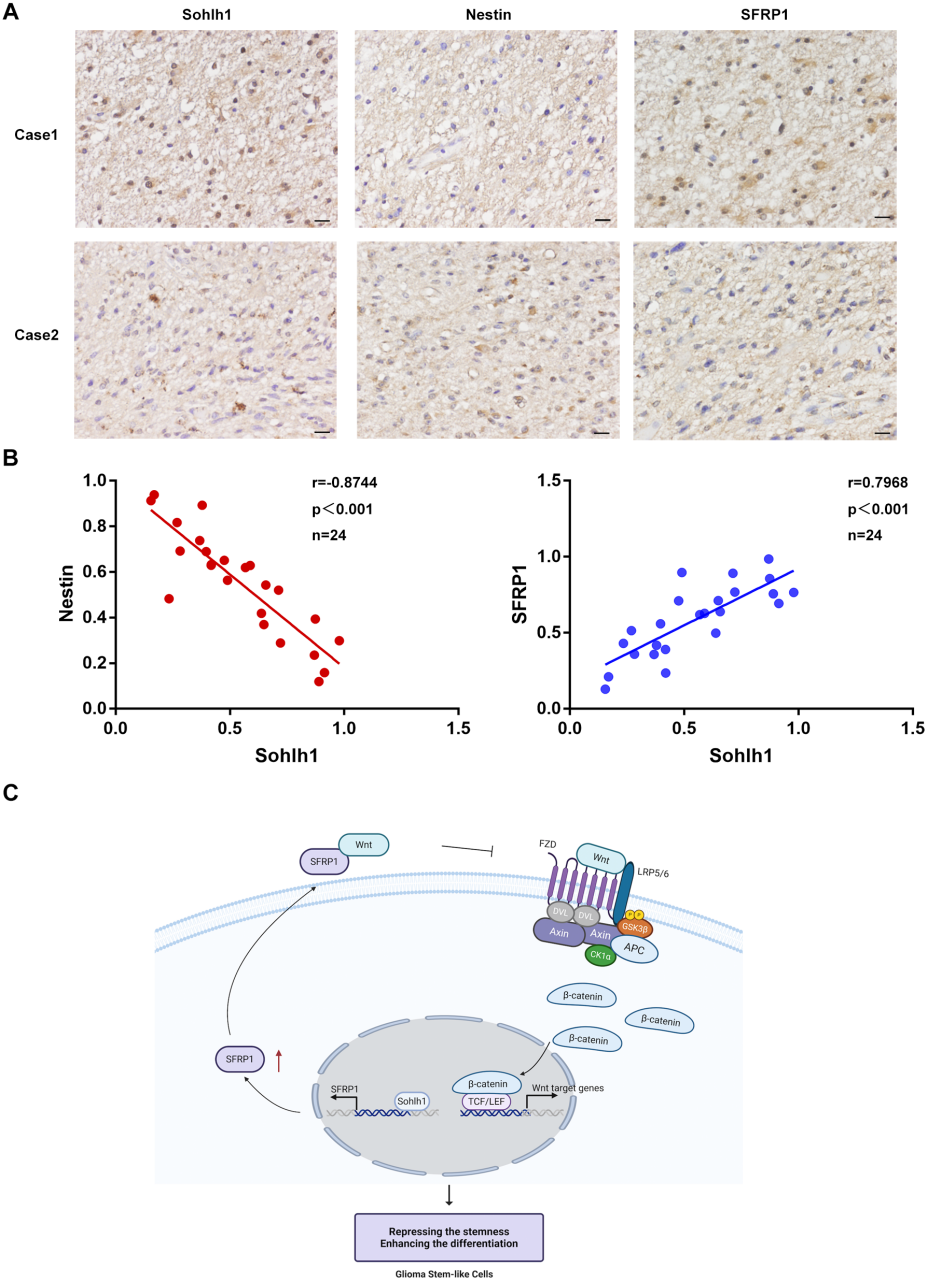
Sohlh1 is negatively correlated with the expression of Nestin and positively correlated with the expression of SFRP1 in glioma tissues. (A) Representative IHC images of Sohlh1, nestin, SFRP1 in glioma samples. (B) Correlation analysis of Sohlh1 and Nestin, SFRP1 in glioma clinical samples. Scale bars indicate 20 μm. (C) A schematic model showing that Sohlh1 directly regulates SFRP1 expression and inhibits Wnt/β‐catenin signalling pathway activation, thereby repressing the GSLC stemness and promoting the GSLC differentiation. Statistical results were calculated using a two‐tailed Student's *t* test. *p < 0.05, **p < 0.01, ***p < 0.001.

To identify the roles of Sohlh1 in human glioma, the relevance of Sohlh1 and Nestin, SFRP1 expression was further evaluated in human glioma tissues. Immunohistochemical staining analysis showed that Sohlh1 was negatively correlated with the expression of Nestin and positively correlated with the expression of SFRP1 (Figure 8A,B). The correlation of Sohlh1 and clinical characteristics was shown in Table S2.

In summary, Sohlh1 regulated the stemness and differentiation of GSLCs through the SFRP1/Wnt/β‐catenin signalling pathway (Figure 8C).

## 3 Materials and Methods

### 3.1 GSLC Culture

The human glioma cell lines U87MG and U251 were obtained from the Cell Bank of the Chinese Academy of Sciences (Shanghai, China). GSLCs were cultured in ultra‐low attachment plates (Corning, NY, USA) and in serum‐free DMEM supplemented with B27 (Gibco, NY, USA), 20 ng/mL of epidermal growth factor (PeproTech, NJ, USA), and 20 ng/mL of fibroblast growth factor‐basic (bFGF, PeproTech, NJ, USA). These cells were maintained at 37 °C, with 5% CO_2_. The cell lines were authenticated by short tandem repeat (STR) profiling and tested free of mycoplasma.

### 3.2 Lentiviral and Plasmid Transfection

To obtain cells stably expressing Sohlh1, we infected GSLCs with Sohlh1 overexpression lentivirus containing the lentiviral vector Luciferase‐GV721 (Genechem, Shanghai, China). After 48 h infection, these cells were cultured in DMEM containing 1 μg/mL puromycin to screen for cells stably expressing Sohlh1. For siRNA experiments, the scrambled shRNA sequences targeting the Sohlh1 gene were cloned into a Luciferase‐GV112 lentiviral vector, and the lentivirus encoding Sohlh1 shRNAs (Genechem, Shanghai, China) was generated and infected cells. As for transient transfections, Lipofectamine 2000 reagent (Invitrogen, CA, USA) was used according to the manufacturer's instructions. The cells were collected after transfection with SFRP1 knockdown/overexpression plasmids for 48 h. Then q‐PCR and Western‐blot assays were used to verify the infection efficiency. The target sequences selected are shown as follows: 5′‐AGGCGTTTCTGGAAAGTCCTT‐3′ (siSohlh1‐1) and 5′‐GTTGACGTTGTCGAGTCAGAT‐3′ (siSohlh1‐2).

### 3.3 Neurosphere Formation Assay

U87MG and U251 GSLC neurospheres were plated in low‐attachment 24‐well plates according to 1 × 10^3^ cells per well, with three replicate wells for each group. After 2 weeks, the number of spheres in each well was counted and photographed with an inverted microscope.

### 3.4 Real‐Time PCR

Total RNA was isolated with TRIzol reagent (Invitrogen, CA, USA) according to the manufacturer's instructions. Reverse transcription reaction (AG, Changsha, China) was carried out using 2‐μg total RNA. Real time PCR (q‐PCR) was performed with Ultra SYBR Mixture (ComWin Biotech, Beijing, China) on a CFX96 Real time PCR Detection System (Bio‐Rad, CA, USA). The PCR primer sequences were displayed in Table S1. Using GAPDH as an internal control for all samples, the relative expression levels of genes are indicated using the $2^{-\Delta \Delta C_{t}}$ method. Each sample was practised in triplicate.

### 3.5 Western Blot

The cells and frozen tumour tissues were prepared and total protein was extracted using RIPA lysate (Solarbio, Beijing, China) containing protease inhibitor cocktails (Sigma‐Aldrich, MO, USA). Protein concentration was measured using the BCA kit (Thermo Fisher Scientific, MA, USA). A total of 50 μg of protein was separated by 12% sodium dodecyl sulphate polyacrylamide gel electrophoresis and transferred to a polyvinylidene fluoride membranes (Millipore Corp, MA, USA). After being closed with 5% nonfat milk powder for 2 h at RT, the membranes were infiltrated in primary antibody overnight at 4°C. This was followed by incubation with peroxidase‐conjugated secondary antibodies (1:5000 dilution) for 2 h at RT. Detection of protein expression was conducted using an ultrasensitive chemiluminescence kit (Thermo Fisher Scientific, MA, USA). The loading amount of sample was calibrated by β‐actin antibody. Primary antibodies are shown as follows: rabbit anti‐Sohlh1 (1:1000, NBP1‐56454; Novus), rabbit anti‐Nestin (1:1000, DF7754; Affinity), rabbit anti‐CD133 (1:1000, AF5120; Affinity), rabbit anti‐CD44 (1:1000, #37259; CST), rabbit anti‐SOX2 (1:1000, #3579; CST), rabbit anti‐OCT4 (1:1000, #2750; CST), rabbit anti‐C‐myc (1:1000, AF6054; Affinity), rabbit anti‐Cyclin D1 (1:1000, AF0931; Affinity), rabbit anti‐MMP9 (1:1000, AF5228; Affinity), rabbit anti‐MAP2 (1:1000, AF4081; Affinity), rabbit anti‐GFAP(1:1000, DF6040; Affinity), rabbit anti‐MBP (1:1000, AF4085; Affinity), rabbit anti‐SFRP1 (1:1000, DF10172; Affinity), rabbit anti‐β‐Actin (1:1000, AF7018; Affinity), rabbit anti‐GAPDH (1:1000, AF7021; Affinity), rabbit anti‐β‐Tubulin (1:1000, AF7010; Affinity).

### 3.6 Immunofluorescence Staining

Immunofluorescence staining was applied to GSLC spheres and tissue sections of xenograft models. The GSLC spheres were fixed with 4% paraformaldehyde for 20 min, and then dropped on the coverslips. The GSLC spheres were washed with PBS, followed by permeabilisation with 0.3% Triton X‐100 for 30 min at RT. The GSLC spheres and tissue sections were stained with primary antibody (anti‐Nestin, CD133, MAP2, GFAP, Ki67, 1:100 dilution) overnight at 4°C and subsequently incubated with red fluorochrome conjugated secondary antibodies for 1 h. Nuclei were stained with DAPI, and images were obtained by fluorescence microscope (Olympus, Japan).

### 3.7 Immunohistochemical Assay

Immunohistochemical assay was applied to human glioma tissue arrays (*n* = 24) and tissue sections of xenograft models. Firstly, the sections were deparaffinised and dehydrated, then incubated in 3% hydrogen peroxide buffer for 30 min. They were boiled in 4% citrate buffer for 15 min. Sections were stained with primary antibodies (anti‐Sohlh1, Nestin, SFRP1, 1:100 dilution) at 4 °C overnight. DAB substrate was used to visualise the antibody binding after incubation with the corresponding secondary antibody for 1 h at room temperature. The images were acquired using an Olympus microscope, and the mean optical density values of each sample were measured by Image‐pro plus software.

### 3.8 Flow Cytometry Assay

GSLCs were collected and washed once with PBS, then resuspended in 100 μL PBS. Added 5 μL of Nestin and CD133 flow‐through antibody (BD, NJ, USA) and incubated at 37°C for 50 min, mixing every 10 min. Finally, the proportion of Nestin and CD133 double‐positive GSLCs was detected by flow cytometry.

### 3.9 Cell Counting Kit‐8 (CCK‐8) Assay

U87MG and U251 GSLCs were seeded in 96‐well plates at a density of 1 × 10^3^ cells per well and incubated with cancer stem‐like cell medium at 37°C for 24, 48, 72, and 96 h. 10 μL CCK8 solution was added and mixed at the same time each day according to the instructions of the CCK8 kit (Solarbio, Beijing, China). After 2 h, The absorbance was measured at 450 nm.

### 3.10 Chromatin Immunoprecipitation (ChIP)

Cells were lysed by ChIP kit (Cell Signalling Technology, MA, USA) to obtain cross‐linked chromatin. Chromatin fragment solutions were incubated with 20 μL of anti‐Sohlh1 antibody or rabbit IgG and protein A/G magnetic beads at 4°C in a rotating state overnight. The precipitated DNA was eluted with a magnetic separator and purified by centrifugal column. q‐PCR and agarose gel electrophoresis were used to analyse. The specific primer pairs for human SFRP1 promoter were as follows: Forward primer 5′‐TTACACCAGCCACGCTGATAA‐3′, Reverse primer 5′‐TAGCAACGGTATTAGCTCTGGG‐3′.

### 3.11 Dual‐Luciferase Reporter Assays

Subcloned the wildtype SFRP1 promoter region (−2000 to −1 bp) with an E box binding site into the pGL3 vector. Sohlh1 overexpression and knockdown GSLCs were grown in 12‐well plates at 5 × 10^4^ per well. The SFRP1 promoter luciferase reporter plasmid was co‐transfected with the pRL‐TK Renilla luciferase plasmid into the target cells using Lipofectamine 2000 (Invitrogen, CA, USA). After 48 h transfection, cells were captured and examined for luciferase activity using the Dual‐Luciferase Reporter Assay System (Promega, WI, USA).

### 3.12 Intracranial Xenograft Experiment

All animal work was carried out in accordance with the protocol approved by the Animal Experimentation Ethics Committee of the School of Basic Medical Sciences, Shandong University. Stably transfected Con and overexpressing Sohlh1 U87MG‐Luc GSLCs (1 × 10^6^/10 μL) were injected into the frontal cortex of the 5‐week‐old male nude mice brain through a hole at a depth of 2, 2 mm to the right of the bregma, and 1 mm posterior to the bregma. After the injection was completed, the needle was left for 3 min and then withdrawn 1 mm every minute. Five mice were used in each experimental group, and nude mice were randomly divided into the control and experimental groups. The phenotype was analysed by a blind investigator. The status of the nude mice was monitored daily. After implantation for 21 days, the animals were photographed with a bioluminescence imaging system (IVIS Lumina II, MA, USA). The brain tissues were sectioned for HE, immunohistochemical, or immunofluorescence analysis.

### 3.13 Statistical Analysis

In this study, GraphPad Prism 7 and Image J software were used to analyse the experimental data. We used the *t* test and *χ* ^2^ test to analyse comparisons between groups. The difference between various groups was considered statistically significant if *p* < 0.05 using the paired *t* test.

## 4 Discussion

Glioma is one of the most lethal tumours. The survival period of glioma patients depends not only on the histological manifestations, resection scope and radiotherapy status, but also on the molecular characteristics including IDH mutation, 1p19g coding deletion, chromosome 10 deletion and PTEN mutation. Most patients have poor prognosis, high recurrence rate and high mortality [30]. GSLCs had been shown to be closely associated with glioma development, progression, treatment resistance and tumour recurrence. GSLCs were one of the first tumour stem‐like cells to be isolated and identified in solid tumours, and in 2003, Singh et al. isolated and identified CD133+ GSLCs with strong self‐renewal capacity from glioma [31]. GSLCs that were isolated from primary glioma or xenografts were able to differentiate into cells with morphological features and markers of neurons, astrocytes, and oligodendrocytes, and these differentiated cells lose their ability to proliferate indefinitely in vitro and in vivo, suggesting that induction of differentiation of GSLCs may be an effective strategy to remove GSLCs [32].

Studies had shown that transcription factors were essential for maintaining the stemness of GSLCs and inducing their differentiation [33, 34]. Sohlh1 and Sohlh2 are both members of the bhlh family of transcription factors and are novel tumour suppressors. Sohlh2 suppressed the proliferation, migration, and invasion of ovarian, breast and colon cancer cells through transcriptional regulation of P21, Cyclin D1 and APC [35, 36]. Sohlh1 inhibited the proliferation, migration and invasion of glioma cells through upregulation of GSK‐3β expression [29]. Sohlh1 may be regulated by DNA methylation (Figure S1). In this study, we found that Sohlh1 was highly reduced in GSLCs, compared to glioma cells (Figure S2). In GSLCs and glioma tissues, Sohlh1 expression was negatively correlated with the expression of GSLC markers, such as Nestin, CD133, CD44, SOX2 and OCT4, whereas Sohlh1 expression was positively correlated with the expression of GSLC differentiation markers, such as MAP2, GFAP and MBP. Ectopic expression of Sohlh1 could restrain the growth of intracranial implanted tumours from GSLCs, whereas Sohlh1 induced the differentiation of GSLCs in vitro and in vivo. The above results indicate that Sohlh1 may serve as an important therapeutic target to eradicate CSLCs in glioma.

Studies had demonstrated that the Wnt/β‐catenin signalling pathway plays a critical role in regulating self‐renewal and differentiation of GSLCs [37, 38, 39]. Aberrant activation of the Wnt/β‐catenin pathway had been identified in a variety of tumours, mainly due to mutations in β‐catenin or other components of the Wnt pathway. Unlike many other tumours, enhanced Wnt signalling activity due to mutations was uncommon in glioma, but dysregulation of the Wnt signalling pathway played an important role in GSLCs [40]. In this study, Sohlh1 reduced the expression of C‐myc, Cyclin D1, MMP2, MMP9 at mRNA and protein levels in GSLCs, which are the downstream target genes of the Wnt/β‐catenin signalling pathway, indicating that Sohlh1 blocks the activation of the Wnt/β‐catenin signalling pathway in GSLCs. In addition, Sohlh1 decreased the expression of β‐catenin in U251 GSLCs nucleus (Figure S3). The addition of Wnt signalling pathway inhibitor LF3 partially blocked the promoting effect of Sohlh1 deficiency on CD133 and Nestin expression in U251 GSLCs (Figure S4). Studies had demonstrated that apoptosis could indirectly reflect the change of Wnt signalling pathway activity [41, 42, 43]. Sohlh1 upregulated the expression of cle‐caspase3 and cle‐PARP in U251 GSLCs (Figure S5). q‐PCR results showed that Sohlh1 upregulated the expression of SFRP1, DKK1 and DKK3, antagonists of the Wnt/β‐catenin signalling pathway in GSLCs, with SFRP1 being the most significantly upregulated, with a more than 10‐fold increase in expression (Figure S6). SFRP1 partially attenuated the roles of Sohlh1 in inactivation of Wnt/β‐catenin signalling, in suppressing GSLC stemness and inducing GSLC differentiation. These results confirmed that Sohlh1 regulates the stemness and differentiation of GSLCs through SFRP1/Wnt/β‐catenin. SFRP1 competitively binds Wnt proteins and prevents Wnt from binding to its receptor. SFRP1 acted as an oncogene and functioned as a tumour suppressor in a variety of tumours. Studies had shown that non‐coding RNA, DNA methylation, allelic imbalance or genomic alterations could affect SFRP1 expression [44], the most common of which is aberrant promoter methylation of SFRP1 [45, 46], but apart from epigenetic regulation, the mechanisms regulating SFRP1 expression in GSLCs were unclear. In our studies, the results of ChIP and dual luciferase reporter assays confirmed that Sohlh1 could directly bind to the promoter of SFRP1 and enhance its transcriptional activity, suggesting that SFRP1 is a direct target of Sohlh1 action.

## 5 Conclusion

In conclusion, our study confirmed that Sohlh1 could suppress GSLC stemness and promote GSLC differentiation in vitro and in vivo. Furthermore, we identified that SFRP1 as a direct target of Sohlh1 mediated the effects of Sohlh1 on GSLC stemness and differentiation. Taken together, our findings suggested that the Sohlh1/SFRP1/Wnt/β‐catenin signalling pathway represented one of the key molecular networks involved in the regulation of GSLC biology in glioma.

## Author Contributions

**Sujuan Zhi:** data curation (equal), formal analysis (equal), writing – original draft (equal). **YanRu Chen:** data curation (equal), formal analysis (equal), writing – original draft (equal). **Yunling Xiao:** project administration (supporting). **Lanlan Liu:** data curation (supporting), investigation (supporting). **Xiaoning Feng:** visualization (supporting). **Xuyue Liu:** visualization (supporting). **Ying Shen:** investigation (supporting). **RuiHong Zhang:** writing – review and editing (equal). **Jing Hao:** writing – review and editing (equal).

## Conflicts of Interest

The authors declare no conflicts of interest.

## Supporting information


Data S1

## Data Availability

Data openly available in a public repository that issues datasets with DOIs.
