# Supplementary material for: Sohlh1 Modulates the Stemness and Differentiation of Glioma Stem‐Like Cells by Inactivation of Wnt/β‐Catenin Signalling Pathway via SFRP1
Source: J Cell Mol Med. 2025 May 26;29(10):e70599. doi: 10.1111/jcmm.70599 (PMC12105582; doi:10.1111/jcmm.70599)
Supplement: Supplementary file 1 — Data S1 [file JCMM-29-e70599-s001.docx]

**Supplementary Table S1: Primer sequence for qPCR**

| Gene | | Forward primer  （5^，^-3^，^） | Reverse primer  （5^，^-3^，^） |
| --- | --- | --- | --- |
| GAPDH | GAAAGCCTGCCGGTGACTAA | | AGGAAAAGCATCACCCGGAG |
| Sohlh1 | AGCTGGGGAATTGGGACTCA | | CAACAGCTAAGCACCAACGG |
| Nestin  CD133  CD44 | AGGAAAAGACCATCTGCCCG  GAGCTAAGGGAAGGGCGG  GATCACCGACAGCACAGACA | | GCCTCTCAGCCAGAAACCAT  TTCTGTCTGAGGCTGGCTTG  GCCTCTTGGTTGCTGTCTCA |
| SOX2 | AGGATAAGTACACGCTGCCC | | TAACTGTCCATGCGCTGGTT |
| OCT4 | AAATAGCACTTCTGTCATGCT | | TATCGAGCACCTTCTATAAGCC |
| MAP2 | CCCCAAGATACAGCTCAGCC | | CTGGGCTCTTGGTTACTCCG |
| GFAP | CCAGCTTGACAGGGAGTGAG | | TGGGAAAATGACGCAGTCCA |
| MBP | GGCAAGGTACCCTGGCTAAA | | TGTACATGTTGCACAGCCCA |
| C-myc | TCATAACGCGCTCTCCAAGT | | CGTTCAGAGCGTGGGATGTT |
| Cyclin D1 | AGCTGTGCATCTACACCGAC | | GAAATCGTGCGGGGTCATTG |
| MMP2 | GTCTGTGTTGTCCAGAGGCA | | CTAGGCCAGCTGGTTGGTTC |
| MMP9 | GTACTCGACCTGTACCAGCG | | AGAAGCCCCACTTCTTGTCG |
| SFRP1 | GGCACAGAGCTGCACTATCA | | CCTCCTCTCTCGGAGACCAA |

**Supplementary Table S2: The correlation of Sohlh1 expression and clinical characteristics**

| Characteristics | Number | Sohlh1 immuno-expression  High level Low level  n=15 n=9 | | r | P-value |
| --- | --- | --- | --- | --- | --- |
| Age(years) |  |  |  |  |  |
| ≤55 | 18 | 12 | 6 | 0.149 | 0.487 |
| >55 | 6 | 3 | 3 |  |  |
|  |  |  |  |  |  |
| Gender |  |  |  |  |  |
| M | 13 | 7 | 6 | 0.175 | 0.414 |
| F | 11 | 4 | 7 |  |  |
|  |  |  |  |  |  |
| Histology Grade |  |  |  |  |  |
| I or II | 11 | 8 | 3 | -0.418 | 0.042 |
| III or IV | 13 | 4 | 9 |  |  |

**Supplementary Table S3**:

Primary antibodies are shown as follows:

| Primary antibodies | Company brand | Dilution |
| --- | --- | --- |
| rabbit anti-Sohlh1 | NBP1-56454; Novus | 1:1000 |
| rabbit anti-Nestin | DF7754; Affinity | 1:1000 |
| rabbit anti-CD133 | AF5120; Affinity | 1:1000 |
| rabbit anti-CD44 | #37259; CST | 1:1000 |
| rabbit anti-SOX2 | #3579; CST | 1:1000 |
| rabbit anti-OCT4 | #2750; CST | 1:1000 |
| rabbit anti-C-myc | AF6054; Affinity | 1:1000 |
| rabbit anti-Cyclin D1 | AF0931; Affinity | 1:1000 |
| rabbit anti-MMP9 | AF5228; Affinity | 1:1000 |
| rabbit anti-MAP2 | AF4081; Affinity | 1:1000 |
| rabbit anti-GFAP | DF6040; Affinity | 1:1000 |
| rabbit anti-MBP | AF4085; Affinity | 1:1000 |
| rabbit anti-SFRP1 | DF10172; Affinity | 1:1000 |
| rabbit anti-β-Actin | AF7018; Affinity | 1:1000 |
| rabbit anti-GAPDH | AF7021; Affinity | 1:1000 |
| rabbit anti-β-Tubulin | AF7010; Affinity | 1:1000 |

**Supplementary Fig S1. Sohlh1 may be regulated by DNA methylation.**

The result of qPCR showed that Sohlh1 expression was significantly up-regulated with 5μM 5-AZA in GSLCs.


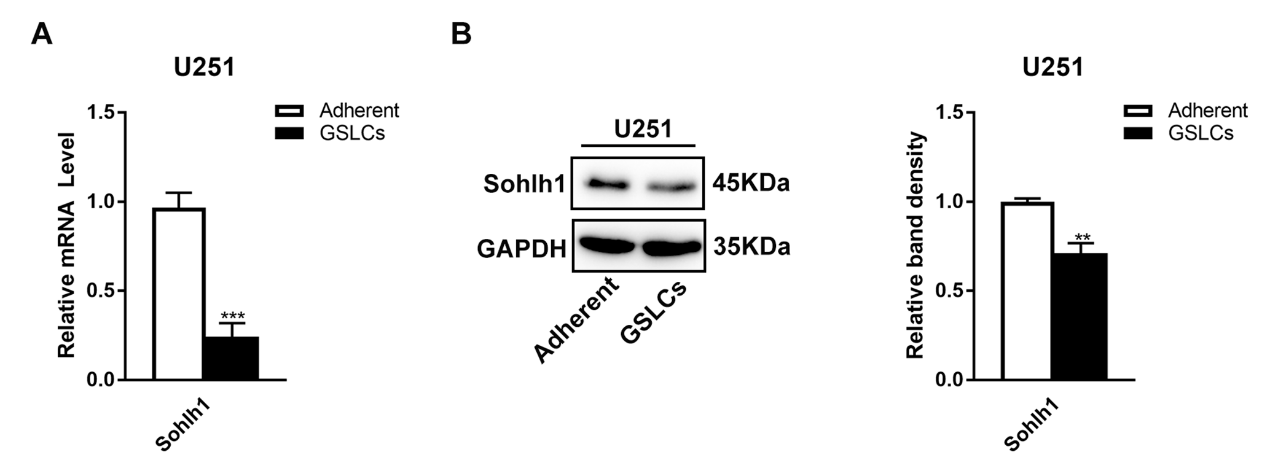
**Supplementary Fig S2. Sohlh1 is downregulated in GSLCs.**

q-PCR (**A**) and Western-blot (**B**) were used to analyze the expression levels of Sohlh1 in adherent cells and GSLCs.

**
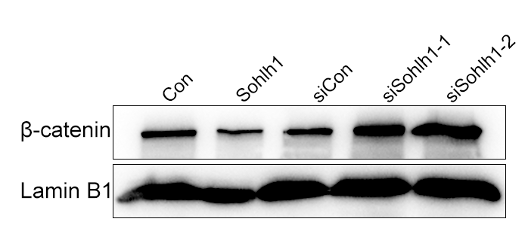
**

**Supplementary Fig S3.** **Sohlh1 decreased the expression of β-catenin in in GSLCs nucleus.**

**Supplementary Fig S4.** **LF3 partially blocked the promoting effect of Sohlh1 deficiency on CD133 and Nestin expression in GSLCs.**

**
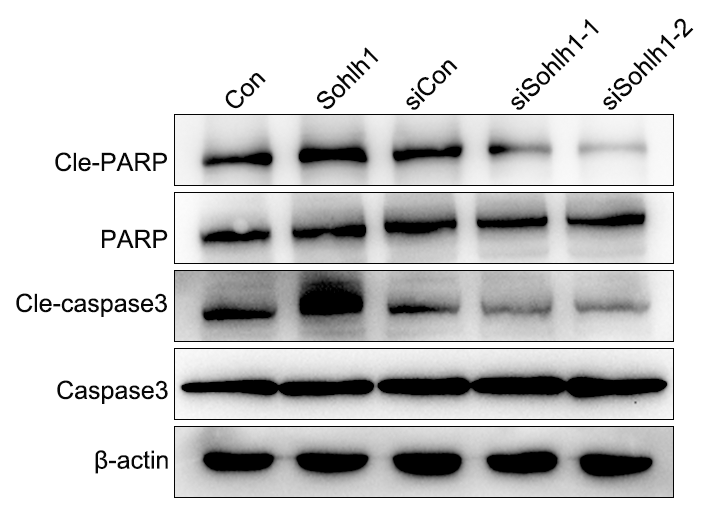
**

**Supplementary Fig S5.** **Sohlh1 up-regulated the expression of cle-caspase3 and cle-PARP in GSLCs.**


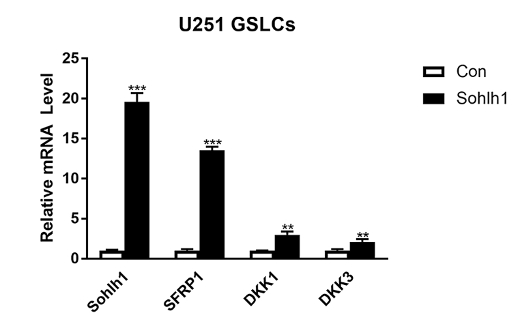


**Supplementary Fig S6. Sohlh1 up-regulated the expression of SFRP1, DKK1, DKK3 in GSLCs.**

Original western blots of Figure 2


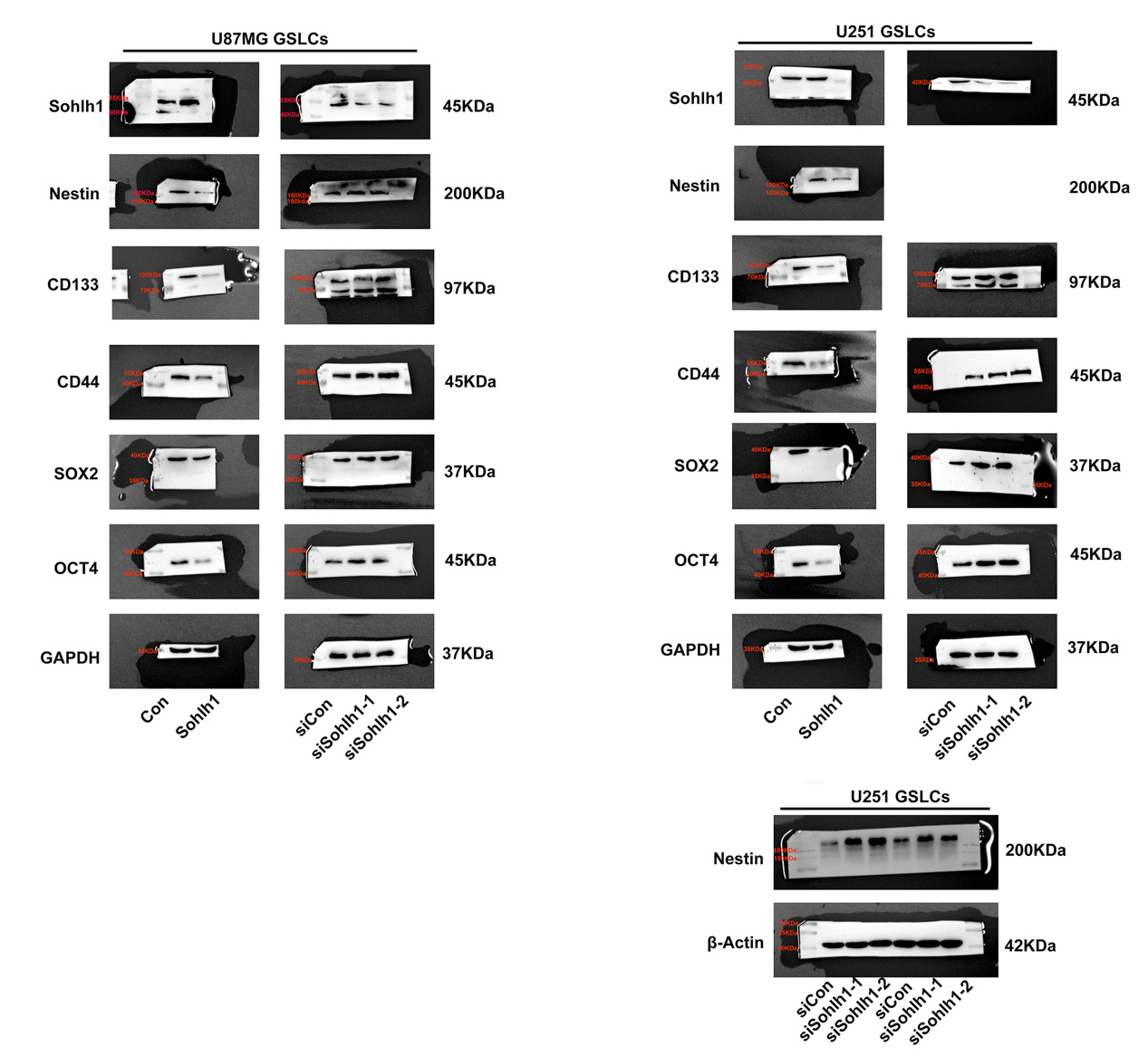


Original western blots of Figure 3


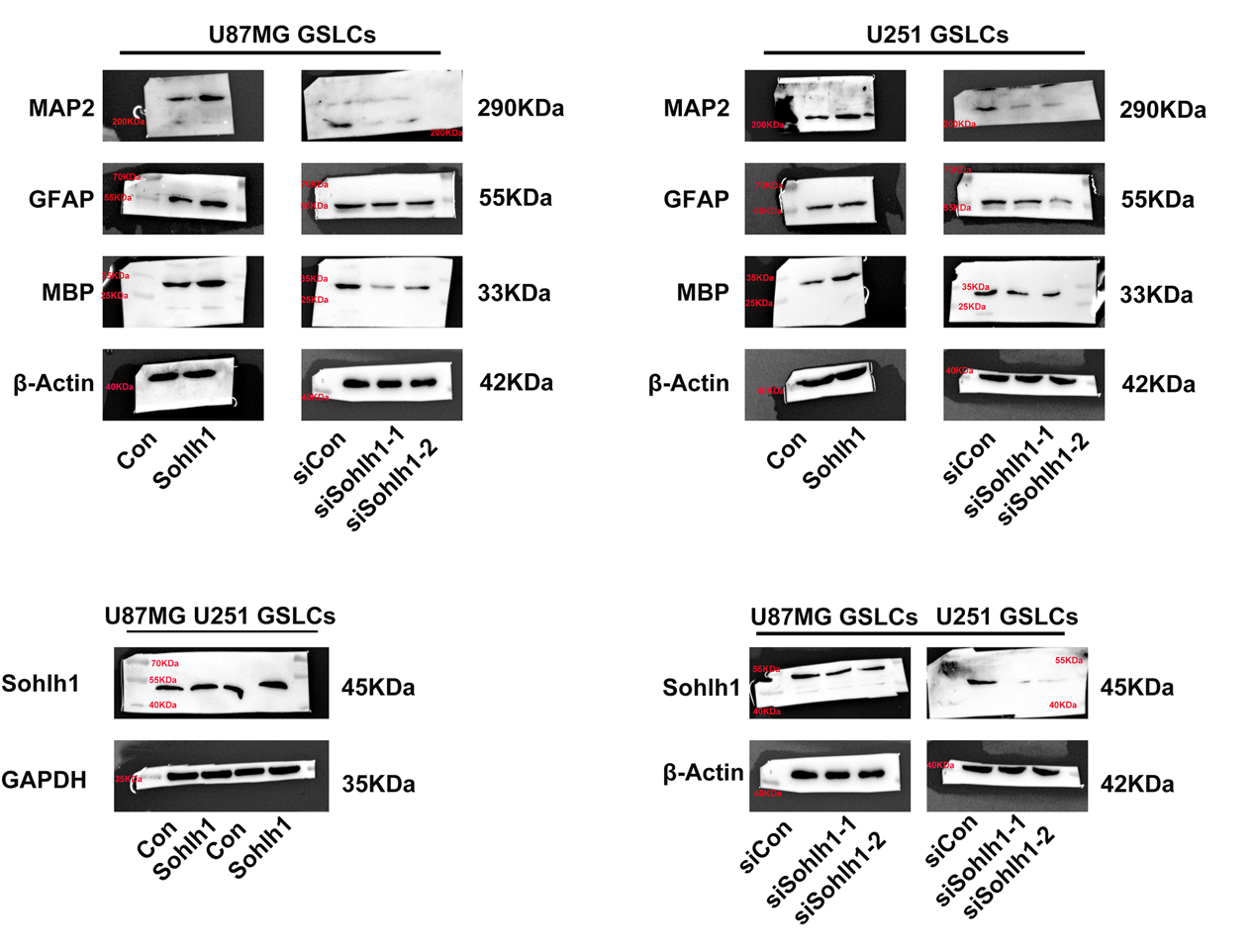


Original western blots of Figure 4


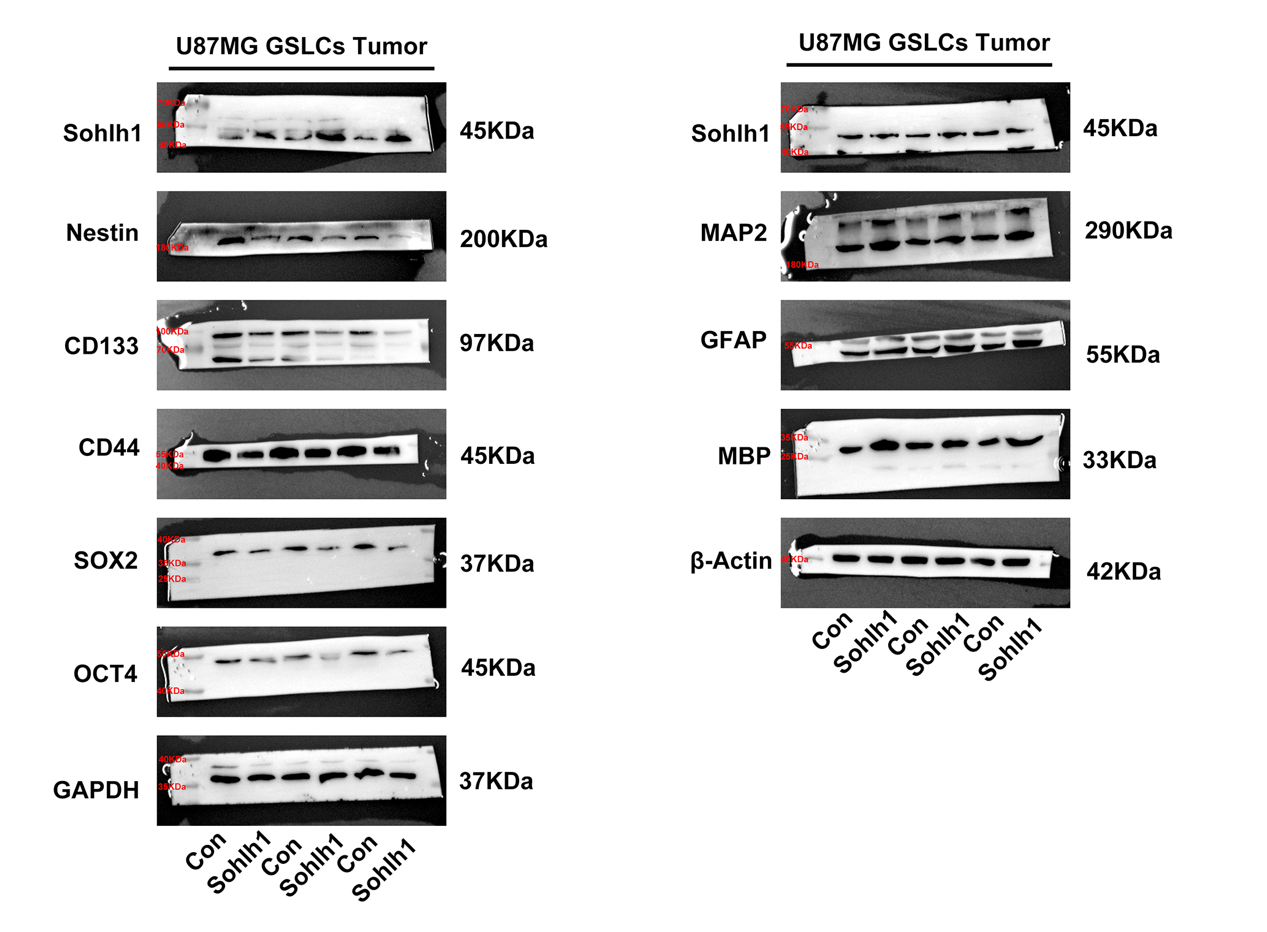


Original western blots of Figure 5


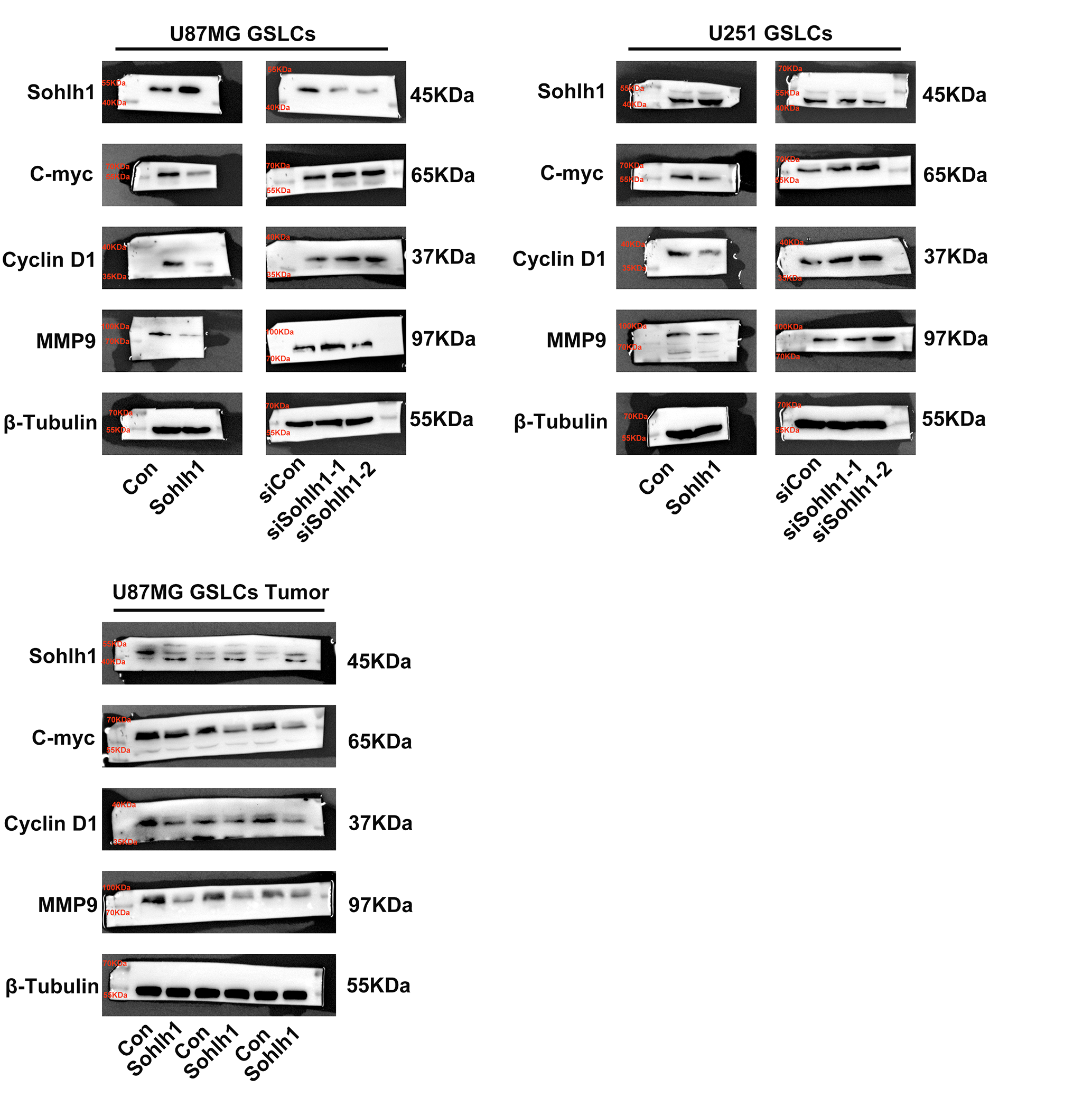


Original western blots of Figure 6


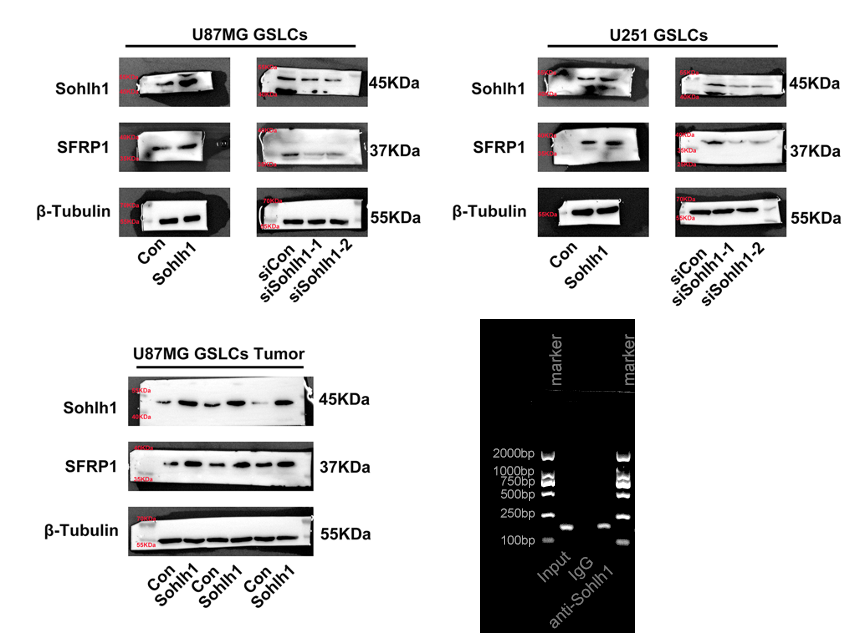


Original western blots of Figure 7


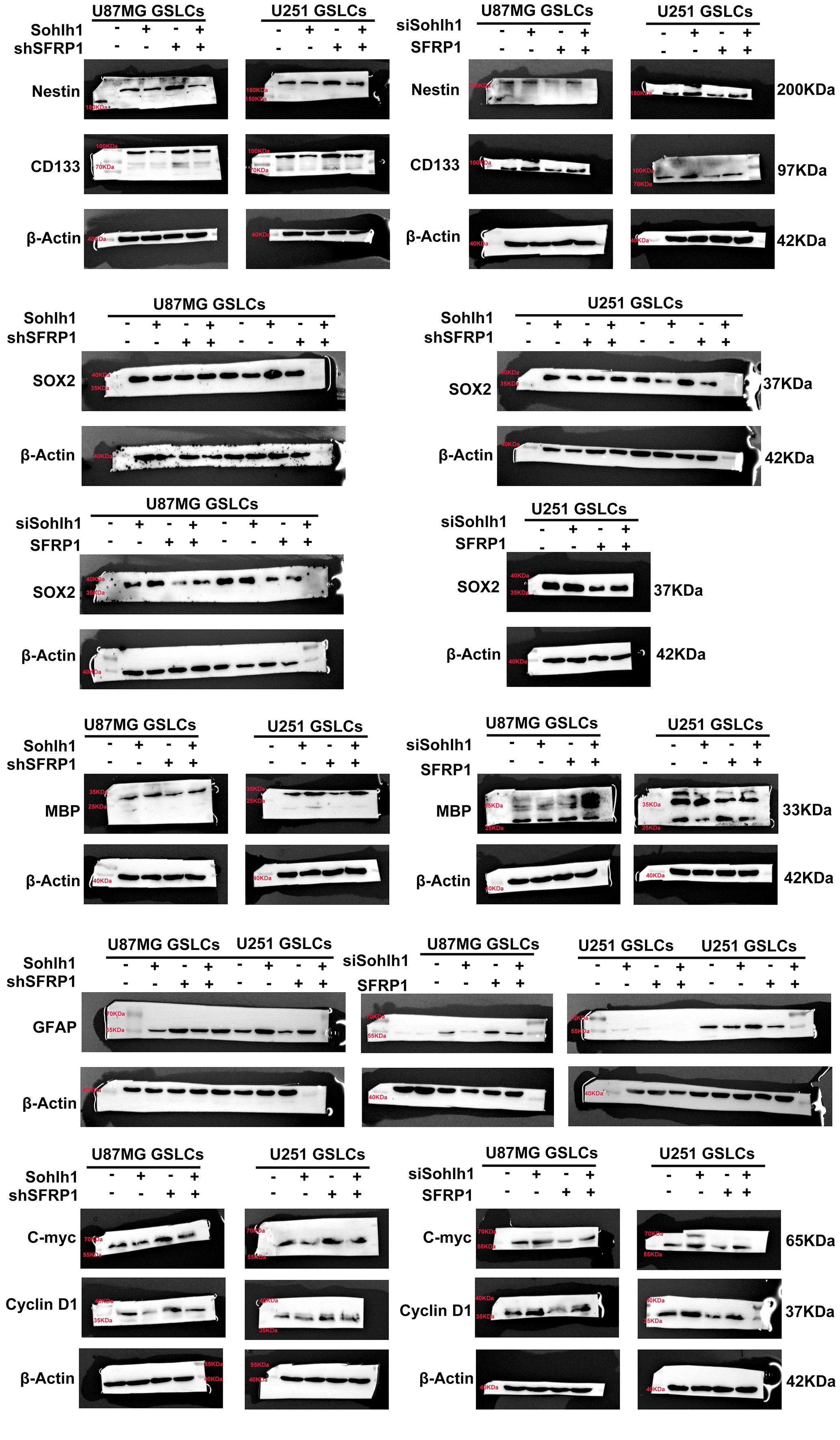


Original western blots of Figure S2


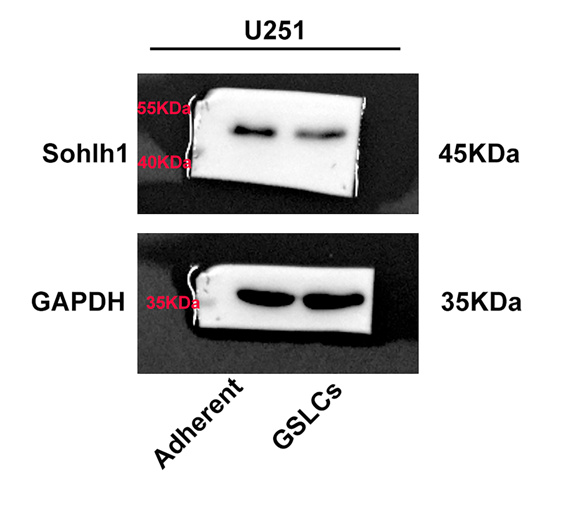


Original western blots of Figure S3

**
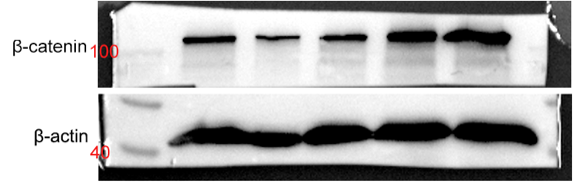
**

Original western blots of Figure S5

**
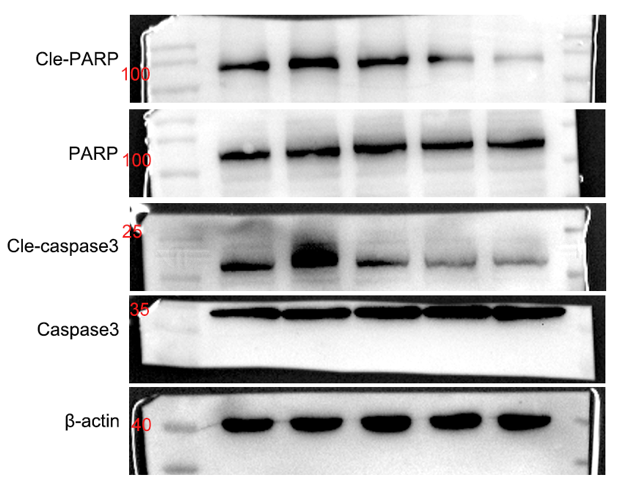
**
